# Accounts of women from Asian ethnic backgrounds about their medical undergraduate experiences in the UK – an interpretative phenomenological study

**DOI:** 10.1186/s12909-025-07181-z

**Published:** 2025-04-21

**Authors:** Aashobanaa Duraisaminathan Valli, Hugh Alberti, Megan Brown

**Affiliations:** 1 School of Medicine, Newcastle University, Newcastle, UK; 2 School of Medicine, Newcastle University, Newcastle-upon-Tyne, UK; 3 Senior Research Associate in Medical Education, School of Medicine, Newcastle University, Newcastle, UK

**Keywords:** Medical education, Discrimination, Asian ethnic minority, Intersectionality, Students support

## Abstract

**Background:**

Asian ethnic women face discrimination within UK medical schools. Their experiences, shaped by intersecting identities, demand focused attention and understanding. This study aims to explore their experiences, especially on understanding how the intersecting identities shape their educational journeys.

**Methods:**

An interpretative phenomenological analysis (IPA) approach was used to explore lived experiences and perceptions of five fourth-year medical students from one institution. Transcripts from semi-structured interviews were analysed following IPA procedures, with an emphasis on understanding the intersectionality of gender and ethnicity in shaping experiences.

**Results:**

Analysis of the transcripts produced four overarching themes with six subthemes. The four themes were: discriminatory action, impact, reaction to the action and expectations and solutions. ‘Discriminatory action’ highlights the direct and indirect discrimination that the students faced. Students’ personal sense of identity shaped the ‘impact’ theme whilst perseverance, tolerance and acceptance of discrimination defined the ‘reaction’ theme. The ‘expectations and solutions’ theme reflects how students anticipate change and propose solutions to mitigate these issues. Participants strongly urged institutions to take more proactive steps in addressing these concerns.

**Conclusion:**

Discrimination significantly impacts the wellbeing and education of Asian women medical students. These challenges have implications for career progression and contribute to broader inequities in medicine. We recommend medical schools consider including a curriculum to address these concerns as a priority in the early years of the medical degree.

**Supplementary Information:**

The online version contains supplementary material available at 10.1186/s12909-025-07181-z.

## Background

There has been a steady rise in the number of ethnic minority students doing a medical degree [1, 2]. According to the UK census data, Asians, the largest ethnic minority group in the UK, comprise of 9.3% of the total population [3], however, 29% of students accepted into medical school are from an Asian background [1]. This is reflective within the NHS population of doctors (33% Asian) including hospital specialists (32%) [4]. A study of junior doctors from ethnic minority backgrounds in the UK found that they experienced psychological distress, fear and felt they were undermined following race and gender related discrimination. This negatively affected their self-esteem and confidence [5].

In the US, where similar studies are predominantly conducted, Asians are often grouped with White populations with Lee et al.l (2023) stating that 57% of Americans feel that Asian Americans are more similar to White Americans than people of colour [6]. In contrast British Asians have historically been racialised, often distinctly seen as perpetual outsiders [7]. Over the years, due to British colonisation in approximately 94 countries [8], British Asians have felt ostracized and isolated from the community, which still occurs to date [9]. Despite this population being perceived as successful [10], they still face racial injustice [9].

Asian women face gender/ethnic stereotyping and discrimination in all workplaces within the UK, often feeling pressured to conceal aspects of their identity to fit into the UK culture. They have fewer opportunities to access quality, equitable career experiences compared to their white counterparts [11]. These are long standing issues that have persisted for decades and are also evident within the South Asian Population [12].

Research indicates that South Asian women in the UK take an average of 4.9 months to secure their first job – 2 months longer than white women, who take 2.8 months [13]. Additionally, 63% of South Asian women report feeling compelled to change their appearance, mannerisms and behaviour to secure, and adapt to, employment [13]. Similarly, numerous studies have reported discrimination against Asian American Women in the workplace [14–17].

The challenges Asian women face in professional environments are reflected in the experiences of Asian students, particularly in competitive fields such as medicine. Despite a significant increase in women applying to medical school, with 64% of medical school acceptances going to women [2], female students from Asian backgrounds regularly encounter discrimination in various forms, ranging from explicit racism to microaggressions [18].

Microaggressions, which are common and often unnoticed, are ‘brief and commonplace daily verbal, behavioural, or environmental indignities, whether intentional or unintentional, that communicate hostile, derogatory or negative slights and insults [19]’. One study found that 70% of their participants (Asian American medical students) faced racial microaggressions and were generally perceived as foreigners. These experiences contributed to burnout and had a negative impact on mental health [20]. Indeed, microaggressions can lead to students struggling with their self-identity and self-esteem, which leads to a loss of confidence [21] and increases the risk of mental health problems such as depression [22]. This identity crisis can result in students feeling invisible, excluded, and as though they do not fit in [23, 24], further reinforcing the structural barriers they face in both education and the workforce.

Microinequities differ from microaggressions in that they involve generalised comments or actions affecting a broader population, whereas microaggressions specifically target individuals based on race, ethnicity, or gender [25, 26]. Both microinequities and the practices of inclusion and exclusion significantly shape individuals’ identities and are subconsciously perpetuated during medical school through the ‘hidden curriculum,’ consisting of unquestioned customs, comments, and behaviours [27, 28]. While microaggressions are common, microinequities – subtle yet persistent acts of exclusion or undervaluation – are reported to be even more frequent in medicine [29]. Though these inequities may seem minor, they can have a profound impact on students’ wellbeing and can cause significant distress that influences personal and professional identities. Navigating these challenges often requires students to adapt or suppress aspects of their identity to fit professional expectations, reinforcing feelings of exclusion and limiting opportunities for representation [29].

A well-documented example of systemic inequities in medical education is differential attainment, where students from ethnic minority backgrounds consistently score lower than their white counterparts in academic assessments [30]. This additional burden, coupled with the inherent stress of medical school, can prevent students from reaching their full potential [19, 31–33]. These challenges can have lasting effects, negatively impacting professionals’ later lives working as physicians – biases can negatively impact productivity, increase the risk of burnout, and ultimately impact the delivery of care to patients [34].

As a theoretical framework, intersectionality provides a nuanced understanding of how different discriminating social factors are interwoven and intersect, relate to, and have effect on one another to influence outcomes [35–43]. Examples of these interconnected factors include gender, ethnicity, power and class. Originating from black feminist theory and critical race theory [41, 44], intersectionality explains how no single factor of someone’s identity can be analysed in isolation. It is commonly applied in fields such as law and sociology [45, 46], and recent research has begun exploring the relevance of intersectionality theory for medical education [17, 27]. Very few studies have explored the experiences of women students from Asian ethnic minoritised backgrounds within the UK [18, 47]. We sought to use an interpretative phenomenological approach (IPA) to understand the lived experiences of women students from Asian ethnically minoritised backgrounds and their perspectives through the lens of intersectionality theory.

## Methods

### Research approach

Interpretivism, which focuses on interpreting human interests and meanings, is the paradigmatic framework for this study. It is typically favoured in qualitative research and medical education [48], as it allows for a deeper understanding of individual participants’ insights rather than striving to develop generalised theories or rules.

### Study design

Phenomenology is a type of qualitative research method which looks at experiences of the participants and develops meanings and understanding behind their lived experiences [49]. The approach to phenomenology used in this study is interpretative phenomenological analysis (IPA), which was developed in psychology, and draws on the philosophical work of Heidegger. Heidegger argued that understanding is always shaped by our prior experiences and that researchers cannot separate themselves entirely from the interpretation process [50]. Rather than conducting a detached analysis, IPA acknowledges that researchers’ own experiences and perspectives are key to interpreting participants’ narratives.

We chose to design our study using IPA to gain a deeper interpretation of how participants’ experiences of their gender and ethnicity intersect to shape their experiences of medical education [31]. While participants may not always be consciously aware of these experiences, IPA integrates both the researcher’s and the participants’ analysis to provide richer detail and deeper meaning to their experiences [51]. Such an interpretative approach has allowed for the lead researcher, who herself is from an Asian background, to draw on her experiences and understanding to shape the interviews. This reflexive approach enhances the interpretation of participants’ narratives, supporting exploration of their experiences in-depth, and with cultural sensitivity [51].

### Setting and participants

We used purposive (specific characteristics required as part of the inclusion criteria) and convenience sampling. Participants were recruited via email, and personal invitation in July and August 2024. Our inclusion criteria focused on women medical students from an Asian background in their fourth year of study at Newcastle, because third years had little clinical environment experience and fifth years had already graduated and so would be difficult to contact for recruitment and sampling. Clinical Students (Year 3–5) were selected for participation over pre-clinical students as pre-clinical students have very little exposure (one day every 12 weeks) to patients. Further, based on the principal researcher’s experiences, microaggressions and microinequities are more pronounced in the clinical years.

### Data collection procedures

Semi-structured interviews were undertaken by the principal researcher (ADV), as is the norm for IPA, to allow students to express their views freely, including sensitive topics, and tell their own stories and opinions in depth. The interview was designed based on the background literature of this project and the principal researcher’s own lived experiences (Table in supplementary material).

All interviews were conducted online through Microsoft Teams due to the study being conducted over the participants’ summer break and because students were geographically located across the UK and abroad. The interviews were recorded and transcribed verbatim.

### Data analysis method

**Fig. 1:**
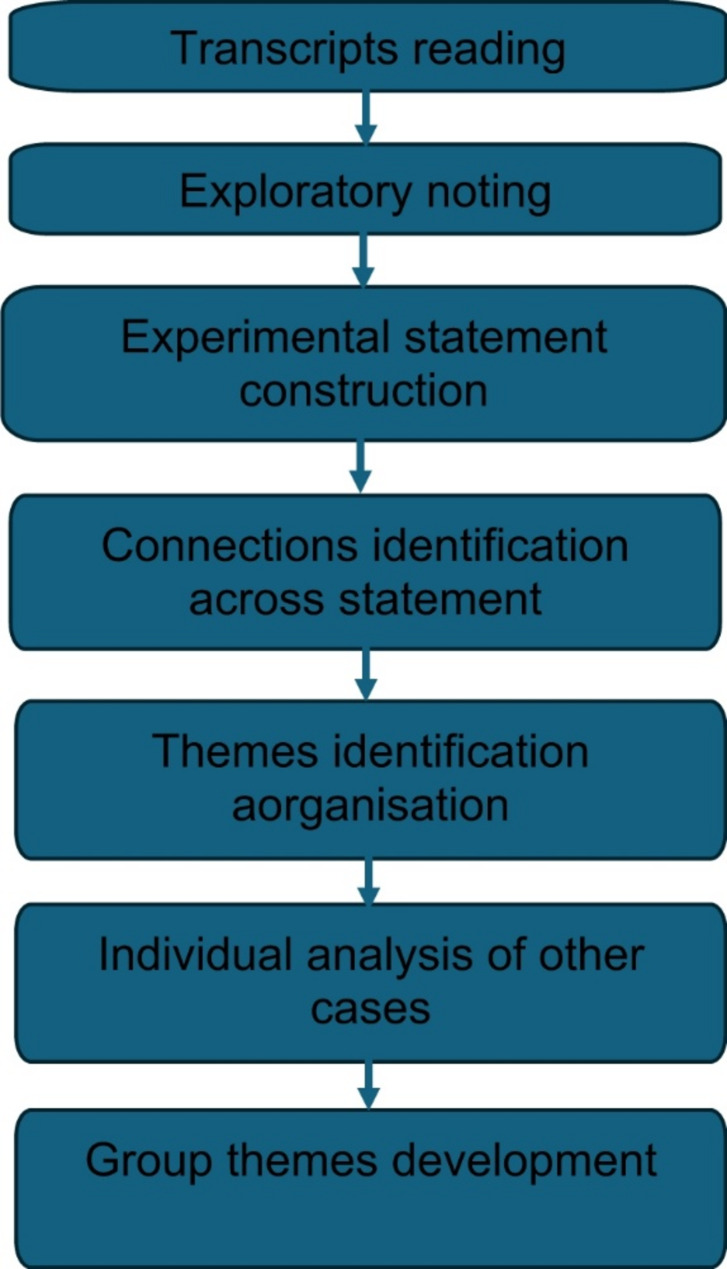
IPA analytical steps

The transcripts were analysed and coded using NVIVO following established IPA analytical steps (Fig. 1) [51]. Coding was done primarily by the principal researcher (ADV) whilst other researchers (HA and MB) contributed to discussions regarding theme creation. All three researchers collaboratively reviewed and refined themes, resolving any discrepancies through discussion. This process also enhanced triangulation, as researchers’ diverse backgrounds provided a broader perspective and deeper understanding of the data.

### Reflexivity

ADV is a medical student from an Asian background herself which helped when assimilating interview questions for the participants. It was also useful when reflecting on their experiences as she was able to understand and relate to the students and was also able to guide the interview to delve deeper into the topic.

HA is a family medicine practitioner (GP), a university employee and a relatively experienced qualitative researcher, who is white and male. MB is a medical education researcher with a PhD in qualitative medical education research, and a strong interest in equality and career progression. The researchers’ diverse research and demographic backgrounds enriched the analysis by offering multiple perspectives and a more comprehensive understanding.

## Results

Five interviews were conducted, lasting between 55 min and 65 min. Four participants were British with a South Asian background and one from East Asia. Students have been referred to using pseudonyms to maintain anonymity but also to maintain the human element to each participant’s stories. Each participant was given the opportunity to choose their own pseudonym with the aim of creating a sense of ownership over their contributions. Analysis of the transcripts provided a total of 30 codes and 4 overarching themes.

The four overarching themes include Discriminatory action, Impact, Reaction, Expectations and Solutions. Discriminatory action highlights the direct and indirect differentiation faced by the students. Students personal sense of identity formed the impact subtheme whilst perseverance alongside their individual tolerance and acceptance of the discrimination comes under the reaction subtheme. Perseverance in this instance focuses on students’ determination to keep pushing forward, and their willingness to work incredibly hard to achieve their long-term goals despite the injustices they face. Expectations and Solutions covers the students’ expectations and their ideas on solutions to mitigate these issues.

The quotes below are taken from the interview transcripts, without altering even grammatical errors, to present the participant voice as it is.

### Theme 1 – discriminatory action

#### Subtheme 1a – direct

Participants described experiencing more severe forms of racism and sexism both in and outside of medical school. Students felt that people discriminating did not understand the severity of their verbal and physical language leading to expressing feelings of fear and insecurity while on placement.

*‘It was in Orthopaedics. It was her [another Asian student] and two other like male colleagues with the consultant. He asked the two boys to go follow him into the consultation room and not her*,* and she thought it was just an accident*,* like he hadn’t pointed out. And then it happened multiple times and then she was like*,* ‘I did not feel comfortable in that situation’.’ – Belinda*.

*‘I think they [a patient] basically just put in a complaint that they thought I was smelly’ – Lucy.*

*‘Sometimes people will just use slurs and don’t think it’s a big deal. I think one that’s quite common is that the P slur’ – Pluto.*

These forms of racism and sexism has led the participants to avoiding certain areas. They perceived them as risky, and showing a preference for staying in urban or city environments where they felt safer.

*‘I know for a fact that I personally would not apply for any rural areas. You don’t know how the neighbourhood might be.’ – Olivia*.

Students noted that not all discrimination is overtly negative; it can be disguised as seemingly positive comments. They felt that many people fail to understand this distinction, which can lead to feelings of discomfort and unease.

*‘Whenever an old man would come in*,* if he needs an ECG and he needs to take his top off*,* he’d make a like comment about one of the girls*,* etcetera. The GP*,* despite being a woman*,* she also kind of reinforced this being like ‘ohh*,* aren’t you lucky to have like 4 girls standing here while you undress’ which makes you uncomfortable*,*’ – Mayan.*

#### Subtheme 1b – indirect

When discussing both ethnicity and gender during their clinical placement years, participants mentioned predominantly facing microaggressions rather than explicit injustice. They felt that these seemingly smaller issues tended to be overlooked; students were not as willing to share openly about their experiences due to being embarrassed or confused with the severity level of the incident.

*‘This happened to in GP*,* I was with a boy [a fellow medical student] and especially with patients as*,* even though I would be doing the consultation*,* they would look at the male colleague and they would be answering to the male colleague.’ – Belinda*.

*‘It’s like it’s so subtle*,* is this just me being sensitive or is it actually should I be feeling weird about it something’ – Olivia.*

On placements, students were often mistaken for someone else, be it related to their gender or the colour of their skin. All students mentioned that they have often been mistaken for nurses and must constantly reiterate and clarify their positions within the team. Despite the mistaken role being an easy mistake to make, students expressed their annoyance at their male counterparts not facing this issue.

*‘There is always one or two patients who will ask*,* ‘oh*,* you are the nurse*,* where is the doctor’*,* that happens quite a lot.’ – Belinda*.

### Theme 2 – impact

#### Subtheme 2a – personal identity

Identity crisis was scattered throughout all the interviews. Many of the students felt that the discrimination they have encountered has significantly affected their confidence. Lucy faced a problem in which a patient had put forward a complaint that she smelled which had a negative impact on her self-worth and subsequently affected her involvement on placement and education. These encounters further intensify feelings of self-doubt, and they were less likely to maximise learning whilst on clinical placement. They also mentioned how they were less likely to contribute answers, and this further worsens their self-esteem.

*‘I did question and doubt myself a bit and I was a bit upset. The next one or two weeks in GP*,* I didn’t examine patients*,* I only took histories as I was a little bit cautious*,* I think.’ – Lucy*.

Frequent thoughts of wishing they were a different ethnicity and gender were expressed by students. Students felt that they would be taken more seriously or be addressed more fairly had they been born a ‘white male’. Students felt that they had to change the way they behaved or have a second face.

*‘I think that I consciously have to change my mannerism’ – Mayan.*

*‘Probably I think you have to change yourself a lot. In terms of food*,* in terms of like everything*,* I think it’s a lot like adjusting like moulding to fit in’ – Belinda.*

*‘I have to be very careful about how I present myself to different people. I feel like I have to choose my manner and my words very*,* very carefully so that I am more palatable*,* almost.’ – Pluto*.

*‘There have been times where you do just think to yourself. If I was a white man*,* this would be so much easier.’ – Pluto*.

Power dynamics play an important part in the students’ response to discrimination. Students felt powerless when a consultant or someone more senior did not condone discriminatory behaviour. They were afraid to attract unwanted attention and felt that it was best to push aside poor behaviour, rather than to act on it.

*‘When your doctor or your boss finds a behaviour acceptable*,* and you are like the lowest of the hierarchy there*,* you don’t want to stand out and be the person that everyone thinks is too sensitive. So*,* if it’s not like active harassment*,* it’s easy to persuade yourself.’ – Mayan*.

This feeling of loneliness is perpetuated when there is nobody to stand up for them, especially fellow peers/colleagues. This worsens insecurity and leads them to question themselves about the situation and whether they are to blame.

*‘I think again like there’s often a case where someone might say something that might be a bit offensive. No one will say anything outright. So*,* if you think it’s wrong*,* you don’t know if everyone else thinks that*,* and if it’s just you overthinking an issue.’ – Mayan*.

Students felt strongly that ‘you are not just representing yourself but also other females of similar backgrounds’ - Mayan. There is a constant fear that making a mistake or doing something wrong would reflect badly on this population. They expressed how they had to prove that they are a worthy and a useful member of the society.

Students felt more comfortable being around friends from the same background. They did not have to constantly explain themselves and there was peer understanding and less judgement.

*‘When I’m with other people of ethnic minorities*,* or even people with the same background as me*,* I know that we have a certain understanding.’ – Pluto*.

Students felt more excited to be on placement when there were role models from a similar race and gender. They felt like they belonged in the hospital. This inspired them and provided them with hope for the future and pride, especially in more male- dominated specialities such as surgery.

*‘I was so excited when I saw her*,* there’s someone who looks like me*,* they’re also female. And that could be someone I can aspire to be.’ – Lucy*.

### Theme 3 – reaction

#### Subtheme 3a – tolerance/acceptance

Throughout our analytical process, we recognised intent as another significant factor [52]. Participants felt that most people they encountered did not have any malicious intent behind any microaggressions and microinequities expressed. Students felt that most comments tended to come from people, particularly the older population, which have not received education on different cultures and backgrounds and instead parroted what they have heard elsewhere.

*‘It comes through*,* but that’s not at least the experiences I have has never been out of malicious intent. It was more just like they’re not educated in that way.’ – Olivia*.

*‘I’ve never had to come across someone who’s done it maliciously*,* so I’ve never really felt offended to be honest.’ – Lucy*.

Participants were a lot more willing to give the public the benefit of the doubt, as patients attending hospitals are in vulnerable positions. Students were able to empathise with the patients and could understand their frustration at being in the hospital.

*‘I think you can’t use it against them because they probably see so many people and they’re sick themselves.’ – Lucy*.

Although students were understanding of the patients’ situations, they were less tolerant and surprised to see discriminatory healthcare staff.

*‘There’s no excuse at all for a doctor to not treat you as well as they can.’ – Mayan*.

Students felt that it was not worth the effort to fight any microaggressions in their short-term placements. Learners found it easier to convince themselves that it is just a one-off minor issue that does not need to be raised further or given too much attention.

*‘It’s almost more of a hassle to deal with than to just put up with it*,* because we are there for a year and it has only been a few times this has happened.’ – Mayan*.

#### Subtheme 3b – perseverance

Opportunities are scarce and students felt that as a female from an ethnic minority background, they had to work a lot harder than their male counterparts to get the same level of opportunities within the clinical environment and to be taken seriously by their seniors and supervisors in the hospital. Although there was a sense of exhaustion and tiredness expressed by students when talking about this topic, there was also a sense of acceptance of this as the status quo and of perseverance despite this norm.

*‘I have heard people say ‘as a female. I must work like 8–9 times as hard’ is really shocking and a bit scary to see’ – Lucy.*

*‘Especially for things like surgery where women in*,* I mean obviously it’s gotten a lot better now compared to previously but there’s always that little bit of thing in the background where for me personally I’m thinking I would probably have to work a bit extra.’ – Olivia*.

### Theme 4 – expectations and solutions

#### Subtheme 4a – expectations

Ethnic minority students wanted equal treatment as everyone else. Many times, participants felt that their student peers, typically white male counterparts, on clinical placement tended to receive more attention and benefitted more from the interaction, both with doctors as well as patients. Students have felt exhausted trying to constantly remind people of their presence. When treated the same, they are a lot happier, and this has a direct positive impact on their learning.

*‘I know what makes me feel a lot better is when I’m being included and treated the same as other medical students’ – Pluto.*

Participants admitted that a more diverse placement in terms of the healthcare staff created a sense of belonging and they felt more like a part of the team. Less diverse settings caused feelings of isolation and alienated the students.

‘By showing that a lot of specialties are diverse, I still feel like as someone from the background. It’s really nice to see.’ – Mayan.

#### Subtheme 4b – solutions

Students suggested a few solutions based on their experiences that could mitigate this discrimination and increase awareness. One student suggested seminar or teaching to increase cultural and religious awareness for students alongside EDI training.

*‘I think it is just taking the time to educate yourself*,* taking the time to look into different religions*,* look into different cultures and just try and understand why people are acting in a certain way.’ – Pluto*.

A couple of students suggested an anonymised app service for people to share their experiences for relatability as well to make the university aware of these incidences for support to be provided.

*‘App or like pathway that students can really raise the issue if they do come across it’ – Olivia.*

*‘I think doing stuff where you kind of ask what people’s perceptions and like kind of like release it back to the year group like knowing that other people feel the same way that I do.’ – Mayan*.

The other major suggestion included transparency of the incident pathway. Many of the students mentioned that when they complained, the situation was fixed and diffused but they felt that there was not much clarity or openness around how the situation was handled.

*‘I think maybe the GP could have*,* I don’t know what she exactly said to them*,* but she didn’t tell me. But she kind of more just told me what had happened and asked whether I needed some support.’ – Lucy*.

*‘I think it would have been quite good if they had told us what happened with the consultant*,* though. Like what they had said to him*,* just so like it feels it makes you feel more validated if they say what’s happened and how that complaint has affected them.’ – Belinda*.

**Fig. 2:**
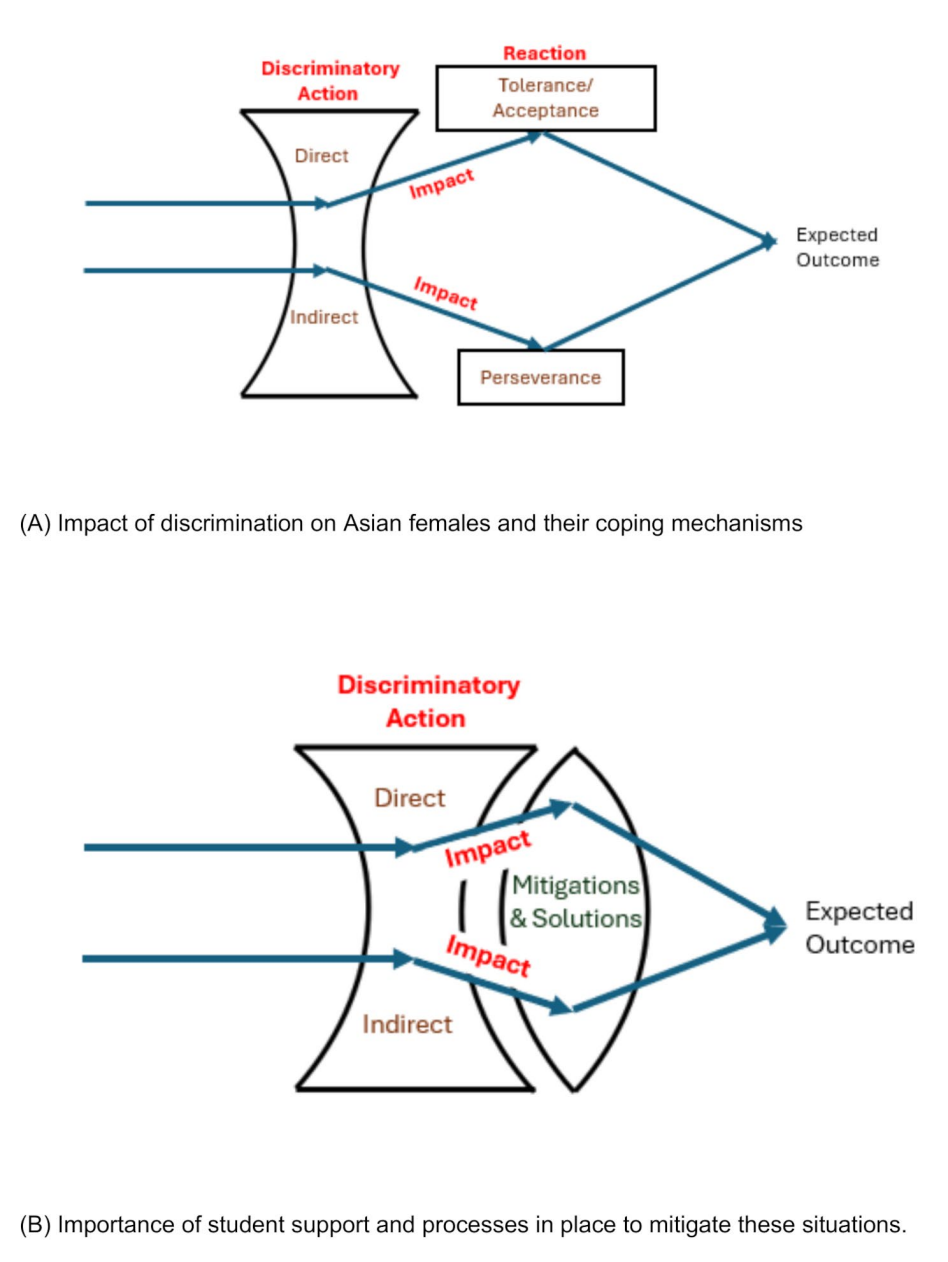
Overarching themes linkage

The themes and the subthemes are summarised in Fig. 2.

## Discussion

Our phenomenological study has explored the effects of racial and gender inequality on the lived experience of women medical students from Asian backgrounds during their time in medical school. The results highlight four overarching themes (Fig. 2) that are further discussed, drawing on wider medical education literature, through the lens of intersectionality theory. In short, this study shows how Asian women in medical school face not only racial discrimination but also gendered injustice that distinctly shapes their experiences – for example, receiving offhanded comments about their appearance, or body odour (racial stereotype). They are also expected to be ‘nurturing’ and are, therefore, perceived as less suited to more competitive specialities within medicine (gender stereotype).

Intersectionality theory emphasises how various demographic factors intersect to shape individuals’ lived experiences; no single factor of a person’s identity can be explored in isolation [15–22]. When asked about both gender and race together, most students tended to focus on either race or gender when describing their medical school experiences, rather than addressing both in an explicitly intersectional and interconnected manner. This may be because the perceived source of discrimination was shaped by the specific context in which that discrimination was experienced – for example, during surgical placements, where gender bias is well-documented [53], students were more attuned to gender-based challenges, whereas in rural GP placements, they were more aware of racial discrimination. The region where this medical school is situated has the highest white population in England, at 90.6% [54], which likely influenced students’ experiences of racial identity in these settings.

While most students compartmentalised their experiences, some explicitly acknowledged the intersection of race and gender, as reflected in the aspiration to be ‘a white male.’ They also reported how collectively and cumulatively microaggressions, related to both race and gender, had a significant impact on their wellbeing and studies. All students mentioned that their experiences negatively affected their self-esteem, amplifying feelings of self-doubt, and in turn, reduced their engagement with patients during placements. A previous study with a similar population focused purely on ethnicity from a constructivist view [48]. Constructivist theory describes how participants construct their own realities based on personal experiences, whereas intersectionality focuses on the intersection between different social identities to shape experiences [35, 36, 55]. Our study highlights the struggles that Asian female students face through both ethnicity and gender which can have a significant impact on their medical education. The focus group discussions (FGDs) of Morrisons et al. (2023) [48] were dominated by the black population (76%), whereas our study is based on the individual experiences of Asian women.

Intersectionality offers us a more nuanced framework for understanding Asian women’s experiences in medical school by capturing the cumulative and interconnected nature of discrimination, offering deeper insight into how both racial and gendered biases influence lived experiences. Indeed, this is the first study in the UK looking at this particular topic through the lens of intersectionality.

Our findings highlight the predominance of stereotypes, which underlie the microaggressions faced by Asian women medical students. These oversimplified beliefs are still extensive and widespread in medicine [56]. Although many stereotypes are widely accepted as negative, research has also identified implicit, unconscious biases that can shape medical education and, in turn, be unknowingly applied in clinical practice, ultimately affecting patient care [57–59]. Many students reported feeling accustomed to these biases yet unable to express their discomfort due to a lack of support, which concords with findings regarding students’ experiences of bias in medicine elsewhere [60]. It has also been reported previously that many students do not speak up following a perceived act of mistreatment [61], increasing stress and anxiety [61, 62]. This disengagement not only hindered clinical learning but also diminished their ability to perform to their fullest potential, supported by research showing students from ethnically minoritised backgrounds perform worse than their counterparts [9–13]. Short placements, combined with a lack of support and transparency from teaching departments at their placements, often leave students unsure about when and how to clearly voice their discomfort and challenges. Longer placements could address these issues and increase comfort levels of students [63, 64].

There is a sense of apathy amongst the students, with many just accepting poor, discriminatory experiences as part of their day-to-day activities of learning that can lead to burnout amongst these students [31] These experiences of microaggressions can have a negative impact on mental health, supported by previous work in Asian American studies [20, 24, 65]. The emotional toll of these experiences, coupled with a lack of time or energy, led many to refrain from reporting incidents, feeling it would not lead to meaningful outcomes. These findings align with studies on Asian Americans, which highlights a reluctance to disclose such situations due to the uncertainties around how to respond appropriately, and fear of shouldering the responsibility alone [20].

Concerningly, the combination of factors we have described that Asian women medical students experience such as frequent microaggressions, lack of support, emotional fatigue and uncertainty not only influences the quality and retention of current education but also future careers. There have been multiple studies that emphasise the impact that bias can have on healthcare staff and ultimately their provision of care to patients [13, 14].

Beyond concerns about discrimination, many participants felt additional pressure to represent their backgrounds and identities, which further widened the gap between ethnically minoritised women and their white male counterparts. This pressure and insecurity can hinder their integration into medicine and clinical teams, potentially leading to feelings of alienation, both within the profession and in broader society, and detrimental to students’ learning, motivation to study and ultimately to personal and professional development [67].

Additionally, invisibility and seclusion – highlighted by our findings – are experiences felt by this population worldwide [20, 23]. As a result, many students question their place in medical school, wondering where they belong and whether they fit in [23]. Participants’ experiences of invisibility and passivity highlight the impact of microinequities in medicine, which differ from the more widely recognised concept of microaggressions. While students encounter both, microinequities are often more subtle, yet still contribute to exclusion [26]. These persistent experiences left participants feeling helpless. Addressing these challenges, particularly during the preclinical and early clinical years, and creating a more inclusive environment would enable students to engage more fully with reduced feelings of isolation and increased sense of community.

A key factor in mitigating these negative experiences is the presence of positive role models. Students reported that positive interactions with mentors and role models increased their likelihood of pursuing similar career pathways, reinforcing the importance of representation and support in medical training. Research has shown that strong role models foster healthy professional development and can help counteract the discouragement that results from discrimination and exclusion [68].

**Fig. 3:**
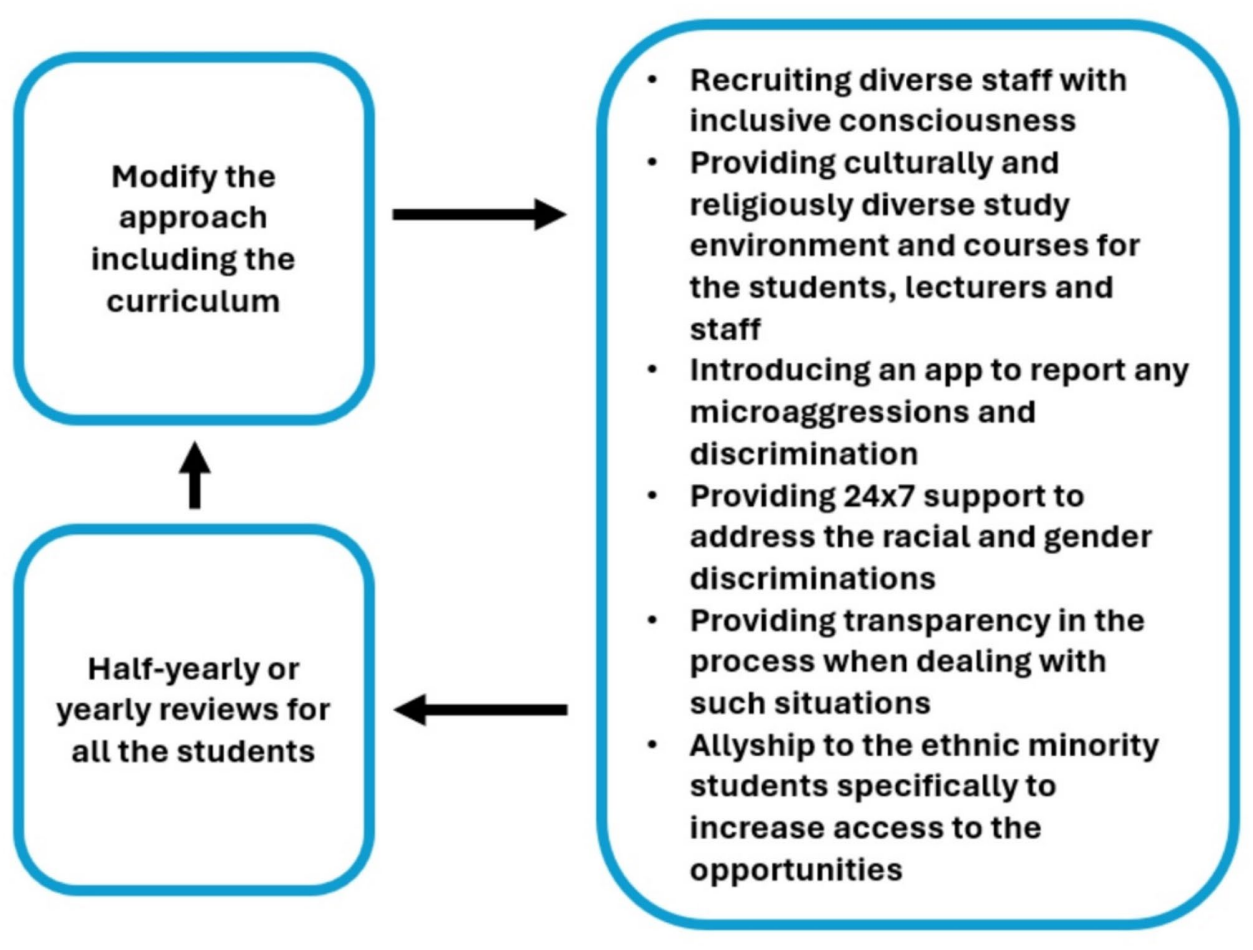
Approach to student support

We have developed several considerations for educators approaching student support, outlined in Fig. 3, based on our participants’ responses. This approach should begin with a thorough assessment of existing student support mechanisms, followed by the introduction of new strategies and measures. These should be continuously monitored and evaluated, with feedback integrated into an evolving, dynamic process. In addition to these recommendations, the approach should incorporate strategies such as promoting positive role models and implementing more longitudinal placements that emphasize supervisor relationships. Further, a secure and supportive platform should be provided for students to anonymously report any incidents.

### Strengths and limitations

Semi-structured interviews using the underlying principles of phenomenology have allowed us to explore in depth students’ lived experiences through the stories they tell about their experiences on clinical placement. The researcher was a female from an ethnic minority background who had an influence on the study, following the guiding principles of interpretive phenomenology. The research was limited to year-4 medical students and to one institution only. We acknowledge that a study involving students from other institutions, and including final years, may provide more holistic, transferable information and there could have been more variety in participant selection, such as to include Black students.

## Conclusion

This study has highlighted that Asian female medical students from ethnically minoritised backgrounds continue to face significant challenges. Racial and gender discrimination both highlight the impact it has on a student’s personal identity, wellbeing and progress, which may ultimately affect their future careers. We have made several recommendations that we hope would be steps towards addressing these issues. This research may be widened spatially and temporally to introduce a new curriculum nationally and to assess the outcome of any curriculum interventions.

## Electronic supplementary material

Below is the link to the electronic supplementary material.

Supplementary Material 1

## Data Availability

Data/Transcripts used and analysed during the study can be made available from the corresponding author on reasonable request.

## Abbreviations

ECG: Electrocardiogram
EDI: Equality, Diversity and Inclusion
GP: General Practitioner
IPA: Interpretative Phenomenology Approach
NVIVO: Non-Versioned Information, Versatile Outcomes
UK: United Kingdom
BAME: Black, Asian and Minority Ethnic
