# Supplementary material for: Accounts of women from Asian ethnic backgrounds about their medical undergraduate experiences in the UK – an interpretative phenomenological study
Source: BMC Med Educ. 2025 Apr 21;25:582. doi: 10.1186/s12909-025-07181-z (PMC12010579; doi:10.1186/s12909-025-07181-z)
Supplement: Supplementary file 1 — Supplementary Material 1 [file 12909_2025_7181_MOESM1_ESM.docx]

Table: Interview Questions

| 1 | Have you ever felt discriminated against in your life, not just in medical school?   - What was this like? - How did it make you feel? - Tell me more on …. |
| --- | --- |
| 2 | Can you tell me an example of when you felt discriminated against due to your race or sex in medical school?   - What was it like when this happened? - How did it make you feel? - Tell me more on … |
| 3 | Have you ever felt proud of your background and when was that?   - Why did it make you feel that way? - What was running through your mind? |
| 4 | Have you ever felt frustrated of your background and when was that?   - Why did it make you feel that way? - What was running through your mind? |
| 5 | Can you tell me an example of when you saw someone discriminate against a colleague?   - What was it like when this happened? - How did it make you feel? - Tell me more on …. |
| 6 | We have talked about discrimination quite heavily, let’s talk about when you felt you were treated equally. Can you give me some examples of this?   - Why did it make you feel that way? - What did the person do in your perspective that made you feel that way? |
| 7 | Have these experiences ever changed the way you think or feel about yourself?   - Do you see yourself differently now than before? - In what ways? |
| 8 | Have these experiences changed where you see yourself in the future as in what specialities or your career progression in the future?   - Why is it? - Can you tell me about that? - What made you feel this way in particular? - What emotions were running through your head when you made this decision? |
